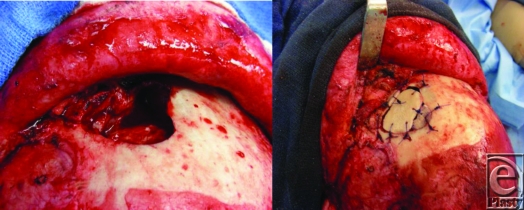# Frontal Sinus Fracture

**Published:** 2011-02-25

**Authors:** Julia Zorn, Richard L. Agag

**Affiliations:** Iona College

## DESCRIPTION

A 27-year-old man with head trauma after a motor vehicle collision.

## QUESTIONS

1. **What are the indications to repair a fracture of the anterior table of the frontal sinus?**
2. **How do you assess injury to the nasofrontal duct by computed tomographic (CT) scan?**
3. **What are the options for obliterating the frontal sinus?**
4. **What are indications to cranialize the frontal sinus?**

## DISCUSSION

Frontal sinus fractures require between 363 kg and 727 kg of force to fracture; therefore, they are usually the result of high velocity impacts, such as car accidents. The frontal sinus has both anterior and posterior tables. Management of frontal sinus fractures is dependent upon the involved tables, the amount of displacement, and involvement of the nasofrontal duct. For isolated anterior table fractures, treatment is only indicated if the fracture is depressed or unstable which would result in a cosmetic deformity if not repaired. Treatment includes either open reduction and internal fixation of the bone fragments or utilization of a titanium plate. Our patient had a lateral anterior table fracture that required Open Reduction Internal Fixation.

Assessment of the nasofrontal duct, which is located posteromedially in the sinus, is paramount for management of frontal sinus fractures. A missed diagnosis of a nasofrontal duct injury can result in mucocele formation. A CT scan with 2 to 3 mm cuts should be done to assess the nasofrontal duct. A fracture through the posteromedial aspect of the frontal sinus is highly suspicious for nasofrontal duct injury and is an indication for operative exploration. Treatment for injury to the nasofrontal duct involves obliteration of the sinus after the mucosa has been meticulously removed. Obliteration can be done with either autogenous or alloplastic materials. In our patient, the nasofrontal duct was not involved on the CT scan that was confirmed intraoperatively during repair of the anterior table. It therefore did not require obliteration.

Cranialization of the frontal sinus is indicated when the posterior table is fractured and displaced greater than one table width. This involves removing the disrupted posterior sinus wall and allowing the brain and dura to expand against the anterior sinus wall. This is done after removal of the nasofrontal duct mucosa and obliteration of the sinus. Although there is a fracture of the posterior table in our patient, it was not more than a table width, and therefore cranialization of the sinus was not indicated.

## Figures and Tables

**Figure:**
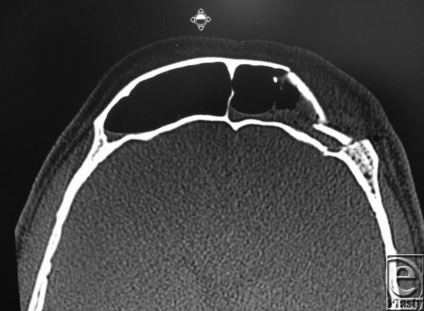


**Figure:**